# Assessment of Accuracy, User Engagement, and Themes of Eating Disorder Content in Social Media Short Videos

**DOI:** 10.1001/jamanetworkopen.2023.8897

**Published:** 2023-04-19

**Authors:** Valerie Lookingbill, Ehsan Mohammadi, Yizhou Cai

**Affiliations:** 1 School of Information Science, University of South Carolina, Columbia; 2 Department of Statistics, University of South Carolina, Columbia

## Abstract

**Importance:**

Social media is simultaneously home to communities of users who promote eating disorders as a lifestyle and users who advocate for recovery. As studies have confirmed an association between exposure to pro–eating disorder content and engaging in disordered eating behaviors, an examination of the accuracy of and interactions with information shared in these complex and contradictory communities can provide insights into content available to users at risk.

**Objective:**

To determine the associations among themes, accuracy of information, and user engagement of eating disorder content on a short video–sharing social media platform.

**Design, Setting, and Participants:**

This qualitative study included a thematic analysis of a sample of 200 TikTok videos, as well as user engagement metrics and content creator characteristics, between February and June 2022. Data were analyzed from March to June 2022.

**Main Outcomes and Measures:**

Content themes, accuracy of information, user engagement, and the associations among these factors were identified in a sample of eating disorder videos on a social media platform. Data were analyzed with Pearson χ^2^, analysis of variance, linear regression, and random permutation tests.

**Results:**

Among the 200 videos assessed, 124 (62.0%) covered prorecovery content, 59 (29.5%) included pro–eating disorder content, and 17 (8.5%) contained anti–eating disorder content. Thematic analysis revealed 4 high-level themes: (1) encouraging the development or sustainment of eating disorders, (2) sharing physical or emotional experiences with eating disorders, (3) sharing narratives of recovery, and (4) social support. While the results of Pearson χ^2^ test indicated that videos in the prorecovery domain contained more accurate content than videos categorized in the pro–eating disorder and anti–eating disorder domains (χ^2^_4_ = 157.92; *P* < .001), the results of analysis of variance demonstrated that there was no significant difference in user engagement between informative and misleading content (likes: *F* = 0.110; *P* = .95; comments: *F* = 2.031; *P* = .13; views: *F* = 0.534; *P* = .59; shares: *F* = 0.691; *P* = .50). Results of the 10 000 random permutation tests, in which all *P* values were between .40 and .60 regardless of the distances, suggested that there was no significant difference in user engagement among the 3 domains.

**Conclusions and Relevance:**

This mixed-methods qualitative analysis of misleading eating disorder information on social media found that pro–eating disorder and prorecovery communities were prevalent. However, social media users in the prorecovery community created more informative than misleading content. Furthermore, the study found no significant difference between users’ engagement with accurate vs misleading videos, which may suggest that false information alone does not influence the virality of videos.

## Key Points

**Question:**

What is the accuracy of eating disorder information disseminated on a video-sharing social media platform and how do users engage with this information?

**Findings:**

This qualitative analysis of 200 videos on TikTok found that prorecovery videos contained more informative content than pro–eating disorder and anti–eating disorder videos.

**Meaning:**

These findings suggest that social media users in the prorecovery community may remediate and address gaps in health information through leveraging technology and experiential knowledge, which could be an effective strategy for building collective resilience against misleading information.

## Teaser

This qualitative study assesses themes of, accuracy of information in, and user engagement with eating disorder content on a short video–sharing social media platform.

## Introduction

TikTok (ByteDance) is a short-form video-sharing social media platform that was one of the most popular social media sites, particularly among adolescents, in the US in 2022.^1^ Recent studies^2,3,4,5^ have demonstrated that users leverage this platform to discuss and offer advice on health-related topics, including eating disorders.^6^ Eating disorders are complex psychophysiological illnesses with the highest mortality rate of any psychiatric disorder.^7^ Eating disorders are most prevalent among adolescent populations^8^ and continue to evolve as a public health threat. The unique features and popularity of TikTok among young generations, compared with other social media platforms, give it potential for facilitating health communication.^9^ However, it also risks spreading misleading information that can lead to negative health outcomes.

Although previous studies have examined eating disorder content on other social media platforms, such as Twitter,^10,11^ Instagram,^12^ and Tumblr,^13^ there has been little systematic study about eating disorder content on TikTok. Studies extended to TikTok have highlighted both the benefits^6^ and risks^14,15^ associated with exposure to eating disorder content and communities on the platform. However, as studies have not fully evaluated the accuracy of the content to which users are exposed, a more comprehensive understanding of the information environment on TikTok is needed to bridge these studies’ findings and identify the platform and content characteristics that enable or impede the dissemination of misleading information. Deeper insight of the information environment on this platform could better inform public health interventions aimed at young and at-risk populations.^16,17^

This study aims to identify the themes present in eating disorder content on TikTok and explore the associations between the accuracy of information and these different content themes. This research also sheds light on the associations of determinant factors of user engagement (ie, views, likes, comments, and shares) with content creator characteristics (ie, number of followers and total likes), content themes, and accuracy of information.

## Methods

This qualitative analysis was deemed exempt from the need for approval and informed consent by the University of South Carolina institutional review board because it did not qualify as human participants research, as videos on the platform were publicly available. This mixed-methods study followed the Standards for Reporting Qualitative Research (SRQR) reporting guideline.

### Study Design and Data Collection

We identified 10 popular hashtags related to eating disorder communities on social media in February 2022. Hashtags act as information anchors^18^ for online communities to discover, disseminate, and gatekeep information and represent a shared meaning that can highlight salient aspects of a topic.^19^ Popular hashtags can identify focal points of a subject and be used as effective tools for social media data collection,^20^ as evidenced by previous health studies.^21,22^ We selected hashtags representing pro–eating disorder and prorecovery communities on TikTok used in previous studies.^6,23^ Additionally, we searched for hashtags identified in similar studies on other social media platforms^12,24^ and misspelled variations of those hashtags, as it is a common hashtagging practice among eating disorder communities^25^ (eTable 1 in Supplement 1). Using stratified sampling, we downloaded 20 videos from each hashtag from the platform’s searchable content on an account created for the study for a sample size of 200 videos, which is comparable to sample sizes of previous eating disorder^6^ and other health-related studies^5,26^ on this platform. Two members of the research team (V.L. and E.M.) independently transcribed a short description of the visual, audio, and textual components of each video and their captions manually before collaboratively identifying and resolving discrepancies to develop the final descriptions for analysis. Furthermore, we collected user engagement metrics (ie, views, likes, comments, and shares) for each video and characteristics of each video’s creator, including a creator’s total number of followers and total likes on all their content, as displayed on a creator’s profile.

### Data Analysis

We conducted thematic analysis^27^ to inductively code data for salient themes, allowing us to examine and identify emergent patterns within the data. We coded 3 primary units of analysis from the written video descriptions, including visual (ie, creator’s appearance and the actions performed by creator), audio (ie, music or voice narration), and text (ie, captions and written text in videos, including closed captioning), as these 3 units together constitute and contextually describe rich content created on social media, as evidenced by previous thematic analyses of social media videos.^6^ Two members of the research team (V.L. and E.M.) independently conducted open coding of 40 videos selected at random, at which we noted a repetition of themes and solidification of interpreted themes, thus reaching data saturation. Following open coding, we used focused and axial coding to summarize and condense the codebook, allowing once more for the identification of emergent coding patterns and categories to occur (eTable 2 in Supplement 1). We elicited 23 subthemes and clustered conceptually similar subthemes into 4 high-level themes (Table 1). These broader-level themes provide a wider perspective about the content. We tested for intercoder reliability with the finalized codebook using Cohen κ and achieved an appropriate value of 0.78^28^ as the Fleiss guidelines consider Cohen κ values above 0.75 to indicate strong agreement levels.

**Table 1.** Themes of Pro–Eating Disorder, Anti–Eating Disorder, and Prorecovery Content on a Video-Sharing Social Media Application

Theme	Subtheme
Encouraging the development or sustainment of eating disorders: creators promote eating disorders as a lifestyle, ask other users to help the creators sustain their own eating disorder, or actively encourage other users to develop or sustain an eating disorder.	Thinspo or meanspo
	Eating with an eating disorder
	Counting calories
	Dieting
	Food guilt
	WIEIAD
	Losing weight to achieve goal weight
	Exercising to achieve goal weight
Sharing physical and emotional experiences with eating disorders: creators share experiential knowledge on eating disorders to raise awareness of or educate other users about eating disorders. In doing so, the creators share their experiences with living with an eating disorder, including how they developed an eating disorder and some of the physical and emotional symptoms associated with an eating disorder.	Best practices for talking to someone with an eating disorder
	Challenging misconceptions of eating disorders
	Revealing the “unglamorous” side of eating disorders
	Combatting pro–eating disorder content
	Personifying eating disorders
	Sharing onset of eating disorder
	Using humor
Sharing narratives of recovery: creators express their personal experiences with recovering from an eating disorder by sharing advice and words of encouragement, as well as the challenges they face during recovery, to other users living with or recovering from an eating disorder.	Interacting with health care professionals
	Recovery
	Celebrating recovery
	Eating in recovery
	Recovery struggles
Social support: creators provide social support to other users, seek social support from other users, or share their experiences with receiving social support from individuals online and offline.	Providing social support
	Receiving social support
	Seeking social support

Abbreviations: meanspo, mean inspiration (a type of thinspiration that uses harsh, negative, and critical language to inspire or motivate weight loss); thinspo, thinspiration (images or text that promote and glorify extremely thin or underweight bodies as an ideal body type to inspire or motivate weight loss); WIEIAD, what I eat in a day.

We then evaluated the accuracy of video content. Initially, we used the DISCERN instrument,^29^ a robust measurement of the accuracy of written health information. However, as similarly reported in other studies of video-based information,^30,31^ DISCERN was ineffective for our sample due to the short length of videos and because not all videos in our sample focused on treatment. Therefore, we adapted classification criterion developed by clinicians,^32^ in which videos were categorized as informative, proanorexia (ie, misleading), or other, as it proved to be an effective method for analyzing the accuracy of eating disorder information in another video-based platform. To mitigate subjectivity, we also evaluated the accuracy of the content by cross-checking the information across 3 reputable health websites (ie, Mayo Clinic,^33^ MedlinePlus,^34^ and National Institute of Mental Health^35^) and then coding the videos into 3 categories: informative, misleading, and other. Informative content describes health consequences of eating disorders and provides advice on how to recover from them. Misleading content includes videos that promote eating disorders as fashion, a source of beauty, or a lifestyle, and includes tips and strategies for developing and maintaining an eating disorder. Misleading content also includes videos that offer inaccurate advice, including recovery advice, such as diet or exercise suggestions, regardless of users’ intentions. We classified content that did not align with either category as other, including content not related to eating disorders or disordered eating behaviors.

Using categories established in a similar study of eating disorder content on other social media platforms,^13^ we categorized the videos into 1 of 3 domains, including pro–eating disorder, anti–eating disorder, and prorecovery based on the video’s visual, textual, and auditory elements. Pro–eating disorder describes content that depicts a desire to engage in disordered eating behaviors with no apparent desire to recover or depicts no recognition that eating disorders are harmful to one’s health. Anti–eating disorder refers to content that challenges pro–eating disorder mentalities and the sharing of pro–eating disorder material due to concerns about the potential harm it may cause in other users. Prorecovery includes content that encourages or celebrates recovery from an eating disorder. We used these domains because they describe a range of eating disorder–related content shared by social media users and address limitations of previous studies excluding eating disorder content more comprehensively.^13^ Finally, we performed Pearson χ^2^, analysis of variance (ANOVA), linear regression, and random permutation tests to measure differences in information accuracy and user engagement among the 3 domains.

Data were analyzed using R statistical software version 4.2.1 (R Project for Statistical Computing). *P* values were 2-sided, and statistical significance was set at *P* < .05. Data were analyzed from March to June 2022.

## Results

### Content Themes

Among the 200 videos assessed, 124 (62.0%) covered prorecovery content, 59 (29.5%) included pro–eating disorder content, and 17 (8.5%) contained anti–eating disorder content. Thematic analysis of video content resulted in 23 subthemes that clustered into 4 high-level themes, as shown in Table 1 and Table 2. Detailed descriptions of themes and subthemes are provided in eTable 3 in Supplement 1.

**Table 2.** Content Themes and Selected Data

Domain	Theme	Subtheme (selected)	Example video	
			Description	Caption
Pro-ED	Encouraging the development or sustainment of ED	Thinspo or meanspo	A recording of 4 female K-pop performers wearing white blazers and shorts dancing on stage. The overlayed text reads: “Babe, it’s only your fault your thighs are rübb1n9 [rubbing] all the time. C4l0r1€$ [Calories] cannot enter your body witchout [without] your consent <3”^a^	“#ed #thin #skinny #kcals #calories #edtitktok #fancam #fyp #xyzbca #fypシ #kpopfancam #sullyoon #weight #bodygoalz #goalweight #weightloss”
	Encouraging the development or sustainment of ED	What I eat in a day	A screenshot of a calorie-counter app detailing the creator’s meals for the day with overlayed text that reads: “this is what i ate today: 339 cals”. For breakfast, the creator consumed 1 cup of nonfat milk (86 calories). For lunch, the creator consumed 2 large hard-boiled eggs (156 calories), 1 red tomato (33 calories), and 1 rice cake (32 calories). For dinner, the creator consumed 1 rice cake (32 calories).	“I ate a bit under my minimum but it’s okay #weightloss #diet #weight #calories #weightgoal #workout #whatiatetoday #edtitktok #fyp #foryoupage”
Anti-ED	Sharing physical and emotional experiences with ED	Combatting pro-ED content	Duet^a^ of a video in which a White feminine-presenting young adult eats apple slices with overlayed text that reads: “When you spent your teens on thigh gap tumblr and now have to spend your 20s in therapy.” The creator (White feminine-presenting young adult) speaks over the original video by saying, “The chokehold that the 2010s Tumblr thinspo had on me is disgusting. Like, that is the reason I developed disordered eating and was obsessed with being skinny and developed anorexia.”	“who else y’all #antidietculture #foodfreedom #emotionaleating #edrecovery”
Prorecovery	Sharing physical and emotional experiences with eating disorders	Revealing the “unglamorous” side of ED	The video zooms out from the creator’s (White, feminine-presenting young adult) face. The overlayed text reads: “TW: ED the sides of ED nobody talks about.” The lyric, “Forgiving who you are, for what you stand to gain. Just know that if you hide, it doesn’t go away,” from MGMT’s “Little Dark Age” plays in the background. The creator removes her wig as the text changes to “Losing all your hair.”	“TW: ED #edrecovery #eatingdisorder #edtiktok”
	Sharing narratives of recovery	Eating in recovery	The creator (White feminine-presenting young adult) documents an experience with facing a fear food, ice cream. The creator poses with a tub of caramel and vanilla ice cream then shows herself scooping the ice cream into a bowl, with overlayed text that reads “not weighing or closely measuring, just scooping it.” The creator sits down at a table and breathes a deep sigh as she scoops ice cream onto her spoon. She takes a bite, pauses, and then smiles with overlayed text that reads: “recovery is the hardest but best thing.” She then documents herself taking 5 more bites, slightly dancing after each. She finishes the video by showing the empty bowl with overlayed text that reads: “you DO deserve to eat.” Throughout the video, the creator narrates: “Hi, I’m [name redacted]. I’ve been in recovery from anorexia for a few months now, and I’m slowly but surely facing all my fear foods. One that I’ve really avoided for such a long time is ice cream. It was a childhood favorite food, but I haven’t eaten it in years. So tonight, as scared as I was, I ate ice cream. I can’t believe I went so long without tasting it, because it was incredible. I’m coming to a point in my recovery journey that I’m really liking the taste of food, and that feels amazing. I chose the ice cream that young [name redacted] would have really wanted and I would have chosen if I was happy and healthy. Despite generally thinking I could not do it, I did it.”	“i hope this proves that you can do anything. i did that! slightly in shock buuuut also proud of myself <3 #edrecovery #fyp #fearfood #icecream”
	Social support	Providing social support	The creator (White feminine-presenting youth) speaks to the camera, saying, “Hi, I’m doing things you’re afraid of to show you it’s okay.” In the next frame, the creator includes a screenshot of another user’s comment, which reads, “Eating in front of people,” as the creator sits down on the grass and holds up a brown paper bag from a fast-food restaurant. She explains, “I’ve got fries from Five Guys [fast food chain restaurant], and I’m with my friends” as she pans the camera to show 3 of her friends before focusing the camera back on herself. She then reaches into the bag, takes out a fry and eats it, as the overlayed text reads “you can eat around people, you’re strong.”	“Reply to [username redacted] doing things you’re afraid of:))) #fyp #edrecovery #bodyacceptance”

Abbreviations: <3, heart emoticon; シ, smile emoticon; ed or ED, eating disorder; fyp, for you page; K-pop, Korean pop music; meanspo, mean inspiration (a type of thinspiration that uses harsh, negative, and critical language to inspire or motivate weight loss]; thinspo, thinspiration [images or text that promote and glorify extremely thin or underweight bodies as an ideal body type to inspire or motivate weight loss); TW, trigger warning; xyzbca, a popular hashtag that some users perceive to increase views on videos.

^a^ A duet is a video in which a creator responds to another creator in a side-by-side video.

The first theme, *encouraging the development or sustainment of eating disorders,* refers to content that promoted eating disorders as a lifestyle or actively encouraged users to develop or sustain an eating disorder and includes subthemes related to “thinspiration” (ie, images or text that promote and glorify extremely thin or underweight bodies as an ideal body type to inspire or motivate weight loss) and “meanspo” (ie, a type of thinspiration that uses harsh, negative, and critical language to inspire or motivate weight loss) as well as sharing diet and exercise information to promote weight loss. Content commonly documented eating habits, including series of “What I Eat in a Day,” a trend in which users post the meals and snacks they consume throughout the day.

The second theme, *sharing physical or emotional experiences with eating disorders,* describes experiential knowledge shared on the social media platform to raise awareness of or educate other users about eating disorders. Subthemes incorporate challenging misconceptions and detailing physical and emotional symptoms of eating disorders, particularly challenging glamorized or romanticized ideas of eating disorders.

The third theme, *sharing narratives of recovery,* refers to personal experiences with recovering from an eating disorder and includes videos related to both celebrations of recovery and stories of recovery difficulties. Content documented users’ recovery journeys, including milestones with meals and associated emotional tolls.

Finally, the fourth theme, *social support,* describes content in which users provided or sought social support or shared their experiences with receiving social support for their eating disorder both offline and online. Content documented users sharing words of encouragement and sentiments that other users were not alone in their experiences.

### Information Accuracy

There were significant differences in the accuracy of information between pro–eating disorder, anti–eating disorder, and pro-recovery content (Table 3). We selected to run Pearson χ^2^ test instead of ANOVA, as the potential responses were categorical, thus the ANOVA for linear case was not suitable, as it was not continuous data. We derived the summation of the χ^2^ distribution with freedom categories using 3 categories and 3 labels in each category for a freedom dimension of (3 – 1) × (3 – 1) = 4. Using asymptotic distribution, the χ^2^_4_ was 157.918; *P* < .001.

**Table 3.** Comparison of Information Accuracy Among Pro–Eating Disorder, Anti–Eating Disorder, and Prorecovery Content

Category	Videos, No.			Total
	Informative	Misleading	Other	
Pro–eating disorder				
Observed	0	47	12	59
Expected	24.0	13.9	21.1	59.0
Anti–eating disorder				
Observed	5	0	11	16
Expected	6.5	3.8	5.7	16.0
Prorecovery				
Observed	76	0	48	124
Expected	50.5	29.3	44.2	124.0
Total				
Observed	81	71	47	199
Expected	81.0	71.0	47.0	199.0

### User Engagement

The random permutation tests determined no significant differences in user engagement (ie, views, likes, comments, and shares) among pro–eating disorder, anti–eating disorder, and prorecovery content (Table 4). To make our results convincing and stable, we repeated the permutation 10 000 times so that the *P* value would be stable among different experiments and would ensure that all *P* values were under the same convincing level. The *P* values under the different distance functions were between .40 and .60, indicating that the results were stable regardless of how we measured. As all *P* values were greater than .05, there was no significant difference in user engagement between the 3 domains. The upper bounds of the 95% CIs of the random permutation test were empirically estimated, which means that means there may be different values in experiments, but the general performance is still stable.

**Table 4.** Comparison of User Engagement of Pro–Eating Disorder, Anti–Eating Disorder, and Prorecovery Content

Engagement	Repeats, No.	2-Order distance		1-Order distance	
		Function value (95% CI)	*P* value	Function value (95% CI)	*P* value
Likes	10 000	96 788.7 (0-248 845.1)	.59	151 239.9 (0-400 443.3)	.61
Comments	10 000	1106.9 (0-3809.2)	.47	1727.4 (0-6142.9)	.45
Views	10 000	670 613.1 (0-1 365 196.3)	.43	958 782.5 (0-2 168 148)	.48
Shares	10 000	3050.2 (0-18 476.7)	.54	4898.1 (0-30 147.1)	.57

Results of the linear regression model show that there was a significant association between creator characteristics (ie, creators’ total number of followers and total likes on all their content) and user engagement (Table 5). The regression formulas can be found in the eMethods in Supplement 1.

**Table 5.** Association Between Creator Characteristics and User Engagement

Model	Estimate (SE)^a^	*t* value	*P* value
Likes model	0.7502 (0.0114)	65.69	<.001
Comments model	0.6015 (0.0505)	11.904	<.001
Intercept of comments and likes	−2.9031 (0.6464)	−4.491	<.001
Views model	0.6804 (0.0515)	13.199	<.001
Intercept of views and likes	2.6932 (0.6595)	4.083	<.001
Shares model	0.7852 (0.0584)	13.433	<.001
Intercept of shares and likes	−6.0311 (0.7477)	−8.067	<.001

^a^ All engagement and characteristics are in log scale.

Furthermore, we used ANOVA to test for an association between creator characteristics and user engagement among the 3 domains (eTable 4 in Supplement 1). Results demonstrate that domains were not significant factors (*F* = 0.110; *P* = .95) in the association between creator characteristics and user likes model without intercept. Similarly, domains were not significant in the association between creator characteristics and other user engagement metrics, including comments (*F* = 2.031; *P* = .13), views (*F* = 0.534; *P* = .59), and shares (*F* = 0.691; *P* = .50) intercept included models.

## Discussion

### Significant Differences in Content Themes Among Domains

This qualitative analysis’s findings of a significant difference in themes among pro–eating disorder, anti–eating disorder, and prorecovery content on social media suggest that communities in these domains have different intentions in their content creation. Although social media, including TikTok, has been criticized as a place to promote disordered eating,^36^ our findings reflect that prorecovery is the most prevalent domain on this specific platform. The differences in content themes discovered in this study align with previous analyses of eating disorder content on TikTok,^6^ Reddit,^37^ YouTube,^38^ and Tumblr,^13^ in that users are increasingly turning to social media platforms for informational and social support for their eating disorders. While discoverability^39^ or content moderation^40^ may have contributed to this prevalence, the active nature of the prorecovery community suggests that while social media may inadvertently offer a space for the proliferation of pro–eating disorder content, it simultaneously offers a space for prorecovery communities and thus can afford users a platform to share their experiences and foster a greater sense of community. Exposure to prorecovery information may have important influences on the formation of health behavior intentions, as when health information is socially embedded into the flow of everyday life, users may perceive the information as more relevant to their needs and may be more likely to use it in decision-making.^41^ Furthermore, while pro–eating disorder and prorecovery content exists in the same digital space, findings from this study and previous research^12^ suggest that not all users object to recovery and may involve themselves in these online communities, even pro–eating disorder communities, to gain social support and a sense of belonging.

### Prorecovery Domain Contained More Accurate Content

Our findings indicate that videos in the prorecovery domain contained more informative content than videos categorized in the other 2 domains. This result suggests that members of the prorecovery community were engaging in community defensive information practices,^42,43^ where users attempted to remediate and address gaps in health information through leveraging technology and their experiential knowledge, which has been similarly reported in online communities of marginalized populations, such as transgender and nonbinary individuals.^44,45^ A strong sense of community among users may serve as an effective strategy for building collective resilience against misleading information^46^ on social media, as misinformation is participatory in nature because it is shaped by technology, social structures, and human action.^47,48^ By incorporating shared community values and tactics into video content, prorecovery users create and engage with information in ways that have positive associations with the health and well-being of their community. While videos in the pro–eating disorder domain exclusively shared misleading content, as similarly reported on other platforms,^32^ misleading information seems to be shared less frequently than informative content related to prorecovery, at least based on our sample. This finding may be related to the new policy that TikTok implemented to minimize misinformation about eating disorder content.^49^

### Similar User Engagement Across Information Accuracy and Domain

Our analysis found no significant difference in user engagement among pro–eating disorder, anti–eating disorder, and prorecovery videos nor the information accuracy within these domains; thus, users were no more or less likely to engage with informative or misleading content or content within a specific domain. While studies commonly show that misleading information is associated with more user engagement in different health and political contexts on social media platforms,^50,51,52^ our findings challenge previous user engagement studies of eating disorder information on other visually based social media platforms. For instance, one study examining YouTube^32^ found that pro-anorexia videos were favored 3 times more than informative videos and were more highly rated by viewers. These differences in user engagement may be attributed to the unique platform features of TikTok, including the shorter length of videos and the tendency for videos to be geared more toward entertainment and shared experiences rather than education, as suggested by a study of urinary tract infections misinformation on TikTok and YouTube,^31^ which found that TikTok videos with higher measures of user engagement contained less misinformation whereas YouTube videos with greater user engagement contained more misinformation.

### Creator Characteristics Were Significantly Associated With User Engagement

We found that there were significant associations between creator characteristics (ie, number of followers and total likes) and user engagement. Statistical analysis reveals that creators’ number of followers and total likes were associated with video performance in terms of user engagement, suggesting that, similar to other social media platforms, accounts with a greater follower base on TikTok receive higher engagement with content, since creators can amplify their content to larger audiences. This finding aligns with previous research on the association between creator characteristics and user engagement on social media, including Instagram^53^ and Twitter,^54^ which concluded that creator-related factors, particularly the number of followers, play a significant role among factors associated with user engagement and virality. However, findings from our study indicate that there was no significant difference between creator characteristics and user engagement among pro–eating disorder, anti–eating disorder, and prorecovery content, which may be attributed to the recent modifications to TikTok’s algorithm^55^ to recognize and break up patterns of content with the same negative themes to minimize the recommendation of limited types of content that may have a negative influence on users when viewed in a filter bubble, such as extreme dieting.

### Limitations

This study had several limitations. First, the study was limited to searchable content tagged with 1 of 10 hashtags. Coupled with the 1-month data collection period, this study’s findings do not document the full landscape of eating disorder content that is likely on this social media platform. Second, there was potential for incorrect identification of creators’ identities. Most creators in our sample were presumed to hold White, feminine-presenting identities, and as studies have shown that TikTok may suppress marginalized populations, such as Black individuals and individuals who identify as lesbian, gay, bisexual, transgender, queer, or other marginalized sexual or gender identities,^56,57^ our study may not accurately reflect the demographics of the larger communities on the platform. Third, we did not analyze video comments. A deeper analysis of comments might complement or complicate the study’s findings, specifically findings on user engagement.

## Conclusions

The findings of this mixed-model qualitative analysis suggest that, while the existence of misleading pro–eating disorder content on social media should not be ignored, users of this video-sharing social media platform were more invested in creating content focused on promoting eating disorder recovery and were engaging less with misinformation than on other social media platforms.^31,58^ Evidence from this research and prior studies^59^ reflect that this short video–sharing social media platform is a rich and diverse atmosphere that may increase community awareness through the sharing of accurate and positive stories about health issues. As social media is an important tool for disseminating health information, particularly among adolescents, we see this as integral to shifting how health professionals think about communicating health information to young populations. Community-driven initiatives, along with digital media fairness, may be effective strategies for collective action to combat unhealthy aspects of social media, especially for high-risk health issues, such as eating disorders. Thus, health professionals should consider these approaches to leverage social media platforms in ways that legitimize and validate the experiences of users.

## Notes

Supplement 1. **eMethods.** Linear Regression Formulas to Measure Associations Between Creator Characteristics and User Engagement **eTable 1.** Pro–Eating Disorder and Prorecovery Hashtags **eTable 2.** Development of Coding Categories **eTable 3.** Themes and Definitions of Pro–Eating Disorder, Anti–Eating Disorder, and Prorecovery Content **eTable 4.** Comparison of the Associations of Creator Characteristics With User Engagement Among Pro–Eating Disorder, Anti–Eating Disorder, and Prorecovery Content

Supplement 2.
Data Sharing Statement
