# Supplementary material for: Assessment of Accuracy, User Engagement, and Themes of Eating Disorder Content in Social Media Short Videos
Source: JAMA Netw Open. 2023 Apr 19;6(4):e238897. doi: 10.1001/jamanetworkopen.2023.8897 (PMC10116364; doi:10.1001/jamanetworkopen.2023.8897)
Supplement: Supplement 1. — eMethods. Linear Regression Formulas to Measure Associations Between Creator Characteristics and User Engagement eTable 1. Pro–Eating Disorder and Prorecovery Hashtags eTable 2. Development of Coding Categories eTable 3. Themes and Definitions of Pro–Eating Disorder, Anti–Eating Disorder, and Prorecovery Content eTable 4. Comparison of the Associations of Creator Characteristics With User Engagement Among Pro–Eating Disorder, Anti–Eating Disorder, and Prorecovery Content [file jamanetwopen-e238897-s001.pdf]

Supplemental Online Content

Lookingbill V, Mohammadi E, Cai Y. Assessment of accuracy, user engagement, and themes of eating disorder content in social media short videos. *JAMA Netw Open*. 2023;6(4):e238897. doi:10.1001/jamanetworkopen.2023.8897

eMethods. Linear Regression Formulas to Measure Associations Between Creator Characteristics and User Engagement
eTable 1. Pro–Eating Disorder and Prorecovery Hashtags
eTable 2. Development of Coding Categories
eTable 3. Themes and Definitions of Pro–Eating Disorder, Anti–Eating Disorder, and Prorecovery Content
eTable 4. Comparison of the Associations of Creator Characteristics With User Engagement Among Pro–Eating Disorder, Anti–Eating Disorder, and Prorecovery Content

This supplemental material has been provided by the authors to give readers additional information about their work.

© 2023 Lookingbill V et al. *JAMA Network Open.*

eMethods. Linear Regression Formulas to Measure Associations Between Creator Characteristics and User Engagement

Regression formula for Model 1:
log(VideoLikes) = b1*log(TotalLikes)
By transformation, it is equivalent to the form of: VideoLikes = TotalLikes^b1

Regression formula for Model 2:
log(VideoComments) = b0 + b1*log(TotalLikes)
By transformation, it is equivalent to the form of: VideoComments = exp(b0) * TotalLikes^b1

Regression formula for Model 3:
log(VideoViews) = b0 + b1*log(TotalLikes)
By transformation, it is equivalent to the form of: VideoViews = exp(b0) * TotalLikes^b1

Regression formula for Model 4:
log(VideoShares) = b0 + b1*log(TotalLikes)
By transformation, it is equivalent to the form of: VideoShares = exp(b0) * TotalLikes^b1

© 2023 Lookingbill V et al. *JAMA Network Open.*

eTable 1. Pro–Eating Disorder and Prorecovery Hashtags

Community	Hashtag	Views (February 2022)
Pro-Eating Disorder		
	#ed	5,300,000,000
	#edtiktokk	4,900,000
	#edtitktok	7,100,000
	#starving	4,444,000
	#thinspho	457,800
Pro-Recovery		
	#edrecovery	3,500,000,000
	#edawareness	690,900,000
	#edrec0very	391,100,000
	#edawarness	185,500,000
	#prorecovery	87,100,000

© 2023 Lookingbill V et al. *JAMA Network Open.*

eTable 2. Development of Coding Categories

Open Codes (selective)	Focused Codes (selective)	Axial Codes
• Promoting pro-ana content<br>• Body weight	• Thinspiration or meanspo<br>• Losing weight to achieve goal weight	• Encouraging the Development or Sustainment of Eating Disorders
• Communicating about eating disorders<br>• Developing an eating disorder	• Best practices for talking to someone with an eating disorder<br>• Sharing onset of eating disorder	• Sharing Physical and Emotional Experiences with Eating Disorders
• Struggling with recovering from an eating disorder<br>• Sharing treatment stories	• Recovery struggles<br>• Interacting with healthcare professionals	• Sharing Narratives of Recovery
• Offering support to other users recovering from an eating disorder<br>• Asking other users for support in recovering from an eating disorder	• Providing social support<br>• Seeking social support	• Social support

© 2023 Lookingbill V et al. *JAMA Network Open.*

eTable 3. Themes and Definitions of Pro–Eating Disorder, Anti–Eating Disorder, and Prorecovery Content

Theme	Subthemes
1. Encouraging the Development or Sustainment of Eating Disorders<br><br>*Creators promote eating disorders as a lifestyle, ask other TikTok users to help the creators sustain their own eating disorder, or actively encourage other TikTok users to develop or sustain an eating disorder.*	1.1. Thinspiration or meanspo<br>*Creator posts images or text to promote thinness, such as images of appearance-related ideals or insulting language to encourage other users to lose weight*<br><br>1.2. Eating with an eating disorder<br>*Creator discusses food, meals, and/or eating in relation to an eating disorder (e.g., creator describes their safe foods or fear foods)*<br><br>1.2.1. Counting calories<br>*Creator counts calories or notes the calories of a food item (e.g., creator recommends a specific food item to viewers because it contains only 50 calories)*<br><br>1.2.2. Dieting<br>*Creator discusses dieting, diet culture, and/or offers advice on diets (e.g., creator describes their progress on the reverse calorie diet) and includes references to starvation and fasting. This code excludes references to exercising as a strategy to lose weight.*<br><br>1.2.3. Food guilt<br>*Creator discusses feeling guilty about or after eating (e.g., creator states that they feel guilty and disgusting after binging)*<br><br>1.2.4. What I eat in a day (WIEIAD)<br>*Creator shows what they eat throughout the day (e.g., creator shows pictures of the foods they consumed throughout the day). This code excludes WIEIADs in recovery*<br><br>1.3. Losing weight to achieve goal weight<br>*Creator discusses or makes reference to losing weight to achieve their goal weight, including losing weight to achieve an unhealthy body weight (e.g., creator asks what their goal weight should be for someone of their height). This code excludes references to specific eating tactics or strategies (i.e., diets or calorie counting) employed to achieve goal weight (see code, "eating with an eating disorder," and related subcodes)*<br><br>1.3.1. Exercising to achieve goal weight<br>*Creator discusses exercising as a means to lose weight (e.g., creator discusses their workout motivation or describes overexercising), details specific exercises to help lose weight, or references the calories associated with specific exercises (e.g., creator asks how many calories they will burn performing a specific exercise)*

© 2023 Lookingbill V et al. *JAMA Network Open.*

eTable 3. Themes and Definitions of Pro–Eating Disorder, Anti–Eating Disorder, and Prorecovery Content (continued)

Theme	Subthemes
2. Sharing Physical and Emotional Experiences with Eating Disorders<br><br>*Creators share experiential knowledge on eating disorder(s) to raise awareness* of or *educate other TikTok users about eating disorders. In doing so, the creators share their experiences with living with an eating disorder, including how they developed an eating disorder and some of the physical and/or emotional symptoms associated with an eating disorder.*	2.1. Best practices for talking to someone with an eating disorder<br>*Creator discusses what to say (e.g., using affirming language) or what not to say (e.g., telling someone that they're happy to see them "finally" eating) to someone who has an eating disorder*<br><br>2.2. Challenging misconceptions of eating disorders<br>*Creator dispels myths or misconceptions of eating disorders (e.g., creator challenges the misconception that only thin people can suffer from an eating disorder)*<br><br>2.2.1. Revealing the "unglamorous" side of eating disorders<br>*Creator describes the physical and/or emotional symptoms of eating disorders (e.g., creator discusses the side effects of eating disorders that "no one talks about," such as losing hair)*<br><br>2.3. Combatting pro-eating disorder content<br>*Creator is adamantly against pro-eating disorder content or discusses the harms of pro-eating disorder content (e.g., creator challenges content about "foods my ED convinced me were absolutely delicious" by explaining how the content can give viewers with eating disorders more ideas)*<br><br>2.4. Personifying ED<br>*Creator positions their eating disorder as another character or agent in the video by assigning their eating disorder human characteristics (e.g., creator plays their eating disorder as a character in their video and refers to their eating disorder, through text or narration, as a little voice)*<br><br>2.5. Sharing onset of eating disorder<br>*Creator discusses or references how they developed an eating disorder (e.g., creator discusses how they started counting calories when they were 12 years-old)*<br><br>2.6. Using humor<br>*Creator makes a joke about their eating disorder or discusses their eating disorder or situation related to their eating disorder in a joking manner*

© 2023 Lookingbill V et al. *JAMA Network Open.*

eTable 3. Themes and Definitions of Pro–Eating Disorder, Anti–Eating Disorder, and Prorecovery Content (continued)

Theme	Subthemes
3. Sharing Narratives of Recovery<br><br>*Creators express their personal experiences with recovering from an eating disorder by sharing advice and words of encouragement, as well as the challenge(s) they face during recovery, to other TikTok users living with or recovering from an eating disorder.*	3.1. Interacting with healthcare professionals<br>*Creator discusses their experiences and/or interactions in a healthcare setting (e.g., creator relays an experience during their time in an anorexia inpatient facility) and/or with a healthcare professional (e.g., creator relays an experience with their therapist)*<br><br>3.2. Recovery<br>*Creator discusses recovering from or having recovered from an eating disorder (e.g., creator shows items in their household that have helped with recovering from an eating disorder, such as self-help books)*<br><br>3.2.1. Celebrating recovery<br>*Creator celebrates their journey recovering from an eating disorder (e.g., creator shows recovery progress photos)*<br><br>3.2.2. Eating in recovery<br>*Creator shows the food/meals they eat during recovery (e.g., creator eats a fear food). This code excludes WIEIADs, which focus on foods that aid creators in losing weight*<br><br>3.2.3. Recovery struggles<br>*Creator highlights the struggles of recovering from an eating disorder, including the emotional toll (e.g., creator discusses re-learning to eat like a "normal person" or talks about "treatment nostalgia")*
4. Social Support<br><br>*Creators provide social support to other TikTok users, seek social support from other users, or share their experiences with receiving social support from individuals on- and offline.*	4.1. Providing social support<br>*Creator offers social support to other users (e.g., creator offers advice to other users or states that they are available if another user wants to talk to someone)*<br><br>4.2. Receiving social support<br>*Creator references support they have received or are currently receiving from others (e.g., creator relays a story of how a family member would sit down with them and made sure they ate)*<br><br>4.3. Seeking social support<br>*Creator asks or seeks support or advice from peers (e.g., creator asks for accountability with achieving their goal weight or asks for advice on how to face a fear food)*

© 2023 Lookingbill V et al. *JAMA Network Open.*

eTable 4. Comparison of the Associations of Creator Characteristics With User Engagement Among Pro–Eating Disorder, Anti–Eating Disorder, and Prorecovery Content

**Likes Model**						
	Residual df	RSS	df	S	F	p-value
Full Model	198	840.61	2	1.41	0.110	0.95
Selected Model	195	839.20	3			
**Comments Model**						
	Residual df	RSS	df	S	F	p-value
Full Model	197	934.91	2	19.08	2.031	0.13
Selected Model	195	915.84				
**Views Model**						
	Residual df	RSS	df	S	F	p-value
Full Model	195	961.67	2	5.29	0.534	0.59
Selected Model	193	956.38				
**Shares Model**						
	Residual df	RSS	df	S	F	p-value
Full Model	196	1243.5	2	8.80	0.691	0.50
Selected Model	194	1234.7				

© 2023 Lookingbill V et al. *JAMA Network Open.*
