# Supplementary material for: Assessment of Accuracy, User Engagement, and Themes of Eating Disorder Content in Social Media Short Videos
Source: JAMA Netw Open. 2023 Apr 19;6(4):e238897. doi: 10.1001/jamanetworkopen.2023.8897 (PMC10116364; doi:10.1001/jamanetworkopen.2023.8897)
Supplement: Supplement 2. — Data Sharing Statement [file jamanetwopen-e238897-s002.pdf]

Data Sharing Statement

Lookingbill. Assessment of Accuracy, User Engagement, and Themes of Eating Disorder Content in Social Media Short Videos. *JAMA Netw Open*. Published April 19, 2023. doi:10.1001/jamanetworkopen.2023.8897

Data
Data available: Yes
Data types: Data (not involving human participants)
How to access data: Data is available upon request (lookingv@email.sc.edu)
When available: With publication

Supporting Documents
Document types: None

Additional Information
Who can access the data: Anyone requesting the data
Types of analyses: For any purpose
Mechanisms of data availability: With investigator support
